# Superficial band of the quadriceps tendon harvested with a minimally invasive technique provides adequate graft dimensions: a cadaveric study

**DOI:** 10.1051/sicotj/2025037

**Published:** 2025-07-16

**Authors:** Napatpong Thamrongskulsiri, Varachaya Khwanjaipanich, Danaithep Limskul, Thanathep Tanpowpong, Somsak Kuptniratsaikul, Thun Itthipanichpong

**Affiliations:** 1 Sports Medicine Research Group, Faculty of Medicine, Chulalongkorn University 1873 Rama IV Rd Pathumwan 10330 Bangkok Thailand; 2 Department of Anatomy, Faculty of Medicine, Chulalongkorn University 1873 Rama IV Rd Pathumwan 10330 Bangkok Thailand; 3 Department of Orthopaedics, Faculty of Medicine, Chulalongkorn University 1873 Rama IV Rd Pathumwan 10330 Bangkok Thailand; 4 Academic Affairs, Faculty of Medicine, Chulalongkorn University 1873 Rama IV Rd Pathumwan 10330 Bangkok Thailand

**Keywords:** Quadriceps, Autograft, Superficial, Harvest, ACL

## Abstract

*Introduction*: This study explored a minimally invasive technique for harvesting the superficial band of the quadriceps tendon. By using a conventional graft tendon stripper, the procedure aims to obtain the full length of tendon fibers necessary for anterior cruciate ligament (ACL) reconstruction. The study aimed to determine if this technique can produce grafts of sufficient length and diameter. *Methods*: From September to October 2023, we conducted a study using full-body Thiel-embalmed cadavers over 18 years of age without pathology-related alterations in lower limb anatomy. The mid-diameter of the graft was measured at its midpoint, and the peripheral diameter was taken at the ends. The length of the triple-folded graft was measured from end to end. A digital vernier caliper measured the length and mid-diameter, and a graft sizer measured the peripheral diameter. *Results*: Sixteen quadriceps tendon autografts were harvested from 16 knees of 8 cadavers (mean age: 64.7 ± 9.9 years). The minimally invasive harvesting technique yielded a mean graft length of 289.0 ± 10.3 mm before folding, a mean mid-diameter of 9.7 ± 0.7 mm, a mean peripheral diameter of 8.5 ± 0.4 mm, and a mean length of 93.1 ± 4.7 mm after triple folding. Gender-based comparisons showed no significant differences. Correlations between graft dimensions and height were not statistically significant. *Discussion*: The findings of this study indicate that the minimally invasive harvesting of the superficial band of the quadriceps tendon resulted in adequate graft dimension. Gender-based comparisons revealed no statistically significant differences in these dimensions between males and females. Additionally, correlation analysis showed weak to moderate correlations between graft dimensions and height, none of which were statistically significant, indicating no meaningful relationship between height and graft dimensions.

## Introduction

Anterior cruciate ligament (ACL) injuries rank among the most prevalent sports-related injuries [1]. An ACL-deficient knee is prone to recurrent instability, meniscal tears, and the development of osteoarthritis [2, 3]. ACL reconstruction has demonstrated favorable outcomes, with several autograft options available, including hamstring, bone-patellar tendon-bone, and quadriceps autografts, each presenting distinct advantages and disadvantages [4, 5].

The bone-patellar tendon-bone autograft facilitates bone-to-bone fixation; however, it is accompanied by certain disadvantages, including postoperative anterior knee pain, difficulty with kneeling, and the risk of patella fractures [5]. Hamstring tendon autografts are widely favored due to their biomechanical properties, which closely resemble those of bone-patellar tendon-bone autografts in the immediate postoperative period [4]. Additionally, the capability of folding the graft permits the creation of multiple graft fixation methods, when necessary, particularly in techniques involving button loops and interference screws. Nonetheless, hamstring autografts may be associated with increased laxity, femoral tunnel enlargement over time, and a heightened risk of postoperative hamstring weakness or saphenous nerve injury during the harvesting procedure [4, 6–8].

The quadriceps autograft has garnered increased attention in recent years due to its robust strength and optimal size for ACL reconstruction [9, 10], effectively mitigating the disadvantages associated with bone-patellar tendon-bone and hamstring autografts, such as the risk of patellar fractures, anterior knee pain, and saphenous nerve injury. The conventional quadriceps tendon harvesting technique involves the extraction of a single tendon band [11], which limits the range of available fixation methods, particularly as it cannot be folded into the loop of a standard cortical button. Furthermore, the traditional open harvesting procedure may result in heightened donor site pain and more prominent scarring [11]. The authors have reported a minimally invasive technique for harvesting a triple-fold superficial layer of the quadriceps autograft [12]. This approach involves harvesting through a limited incision and removing only the superficial layer of the quadriceps tendon, which may offer superior cosmetic outcomes and maintain quadriceps strength postoperatively. However, the reported minimally invasive harvesting technique has yet to demonstrate its suitability for ACL reconstruction in terms of graft length and diameter.

This study explored a minimally invasive technique for harvesting the superficial band of the quadriceps tendon. By using a conventional graft tendon stripper, the procedure aims to obtain the full length of tendon fibers necessary for ACL reconstruction. The objective of this study was to evaluate whether the minimally invasive technique for harvesting a triple-fold superficial layer of the quadriceps autograft can achieve grafts of sufficient length and diameter. The authors hypothesized that this technique would produce autografts with adequate dimensions for effective ACL reconstruction.

## Materials and methods

### Design and setting

All human specimens utilized in this study were donated through a program at the Faculty of Medicine, Chulalongkorn University, with consent for use in educational and research activities. The donors’ bodies were preserved using Thiel’s embalming technique [13] by the Chulalongkorn University Surgical Training Center. The study was conducted with a commitment to respecting human dignity and received approval from the Institutional Ethics Committee of the Faculty of Medicine, Chulalongkorn University (IRB no. 0702/66).

### Specimens

Sixteen knees from eight full-body Thiel-embalmed cadavers (4 males and 4 females) were included in this study. Only cadavers aged 18 years or older were considered. The exclusion criteria encompassed any pathology-related alterations in lower limb anatomy, including trauma or prior surgical interventions. The study was conducted from September 2023 to October 2023.

### Surgical techniques

Full-body Thiel-embalmed cadavers were utilized. A minimally invasive technique was employed to harvest a superficial layer of the quadriceps autograft, following the previously established surgical technique [12, 14]. The harvesting procedure was conducted by a single fellowship-trained sports medicine surgeon (N.T.).

**Figure 1:**
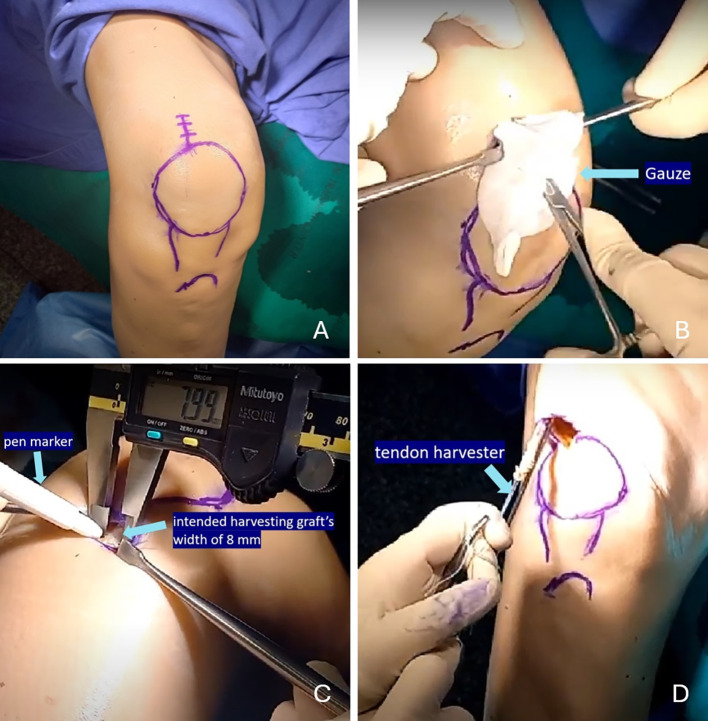
Minimally invasive technique for harvesting a triple-fold superficial layer of the quadriceps autograft from a right cadaveric knee. (A) A 2-cm vertical incision was made over the superior pole of the lateral one-third of the patella for graft harvesting. (B) Gauze was introduced through the incision at the level of the quadriceps tendon to create a soft tissue tunnel between the tendon and the subcutaneous layer. (C) A surgical pen marker was used to delineate the intended graft width of 8 mm. (D) The 8-mm closed-end tendon harvester was employed to strip the graft. Adapted from Arthroscopy Technique, Vol. 12, Thamrongskulsiri N, Limskul D, Tanpowpong T, Kuptniratsaikul S, Itthipanichpong T, Minimally invasive harvesting of triple-fold superficial layer quadriceps autograft for knee ligament reconstruction, pp. e2239–e2246, Copyright Elsevier (2023), with permission [12].

The Thiel-embalmed cadavers were positioned in the supine orientation, with the leg extended and supported on the author’s thighs, away from the operating table. A vertical incision measuring 2 cm was made over the superior pole of the lateral one-third of the patella for graft harvesting (Figure 1A). The subcutaneous tissue was meticulously dissected using Metzenbaum scissors, and the subcutaneous fat was removed with the same instrument to expose the quadriceps tendon. A blunt dissection technique was then used to develop a plane between the subcutaneous layer and the underlying tendon. The gauze was introduced through the incision at the level of the quadriceps tendon to create a soft tissue tunnel between the tendon and the subcutaneous layer (Figure 1B).

A surgical pen marker was employed to delineate the intended graft width of 8 mm, which corresponds to the diameter of the tendon harvester (Arthrex, Naples, FL) (Figure 1C). A No. 15 scalpel blade was used to perform an incision on the lateral one-third of the quadriceps tendon. Careful attention was paid to maintaining the orientation and depth of the cut within the superficial layer of the tendon. A Mosquito clamp was then used to develop a plane approximately 2 cm proximal to its patellar insertion, separating the superficial layer from the deeper tendon structure. This step is critical because the distal quadriceps tendon often has fused layers, complicating graft harvest.

Cord tape was then looped around the detached superficial layer of the quadriceps graft, and Metzenbaum scissors were employed to release the adhesion band connected to the deep layer. The graft was then freed from its attachment at the superior pole of the patella using a No. 15 scalpel blade. It was subsequently sutured using the whipstitch technique with FiberWire (Arthrex, Naples, FL). Following this, the 8-mm tendon closed-end harvester (Arthrex, Naples, FL) was used to strip the graft (Figure 1D). During the harvesting process, the cadaveric knee was extended to reduce quadriceps tension, and the superficial quadriceps graft was harvested through a minimally invasive incision.

The harvested graft was prepared on the operating table. The muscle fiber that attached to the tendon graft was removed using the blunt side of the scalpel. Then the tendon graft was whipstitched on both ends. The graft was measured and divided into three equal segments: proximal, middle, and distal. First, the proximal segment was folded onto the middle segment with the limbs of the whipstitches separated. Next, the distal segment was folded back to the middle segment, and one limb of the whipstitches was passed through a space between the previously folded parts of the graft. Finally, both whipstitches of the graft were pulled to create an equally distributed triple-folded graft [15].

### Outcome measurements

All measurements were conducted by two fellowship-trained sports medicine surgeons (N.T., V.K.). Each measurement was taken twice, with a 15-minute interval between the two measurements. The final values were calculated as the means of the measurements from both investigators and both trials.

**Figure 2:**
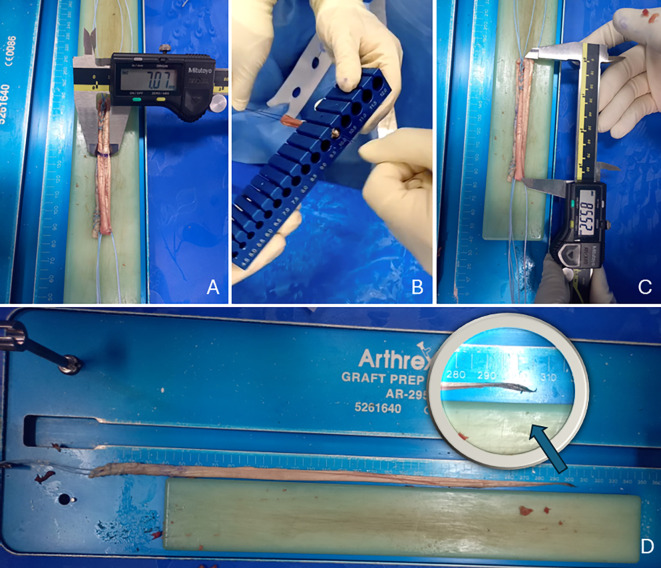
Measurement of the graft: (A) the mid-diameter of the graft measured at the midpoint after triple folding using a digital vernier caliper, (B) the peripheral diameter measured at the end of the graft after triple folding using a graft sizer, and (C) the length of the graft measured from end to end after triple folding using a digital vernier caliper (D) the length of graft before folding. Adapted from Arthroscopy Technique, Vol. 12, Thamrongskulsiri N, Limskul D, Tanpowpong T, Kuptniratsaikul S, Itthipanichpong T, Minimally invasive harvesting of triple-fold superficial layer quadriceps autograft for knee ligament reconstruction, pp. e2239–e2246, Copyright Elsevier (2023), with permission [12].

The graft’s length before folding, mid-diameter after triple folding, peripheral diameter after triple folding, and length after triple folding were measured. The graft’s length before folding was defined as the full length of the harvested graft before folding. The mid-diameter was measured at the midpoint of the triple-folded graft, while the peripheral diameter was taken at the end of the graft. The length of the triple-folded graft was measured from end to end after folding. The graft’s length, mid-diameter, and length after triple folding were measured using a digital vernier caliper (Mitutoyo, Japan). The peripheral diameter of the triple-folded graft was measured using a graft sizer (Arthrex, Naples, FL) (Figure 2).

### Statistical analysis

The statistical analysis was conducted using Jamovi 2.3.28 for Windows. The means of the two measurements were used to evaluate the outcomes. Descriptive statistics are presented as the mean, standard deviation, and range. Gender and graft dimensions were analyzed using the student’s *t*-test, and Pearson’s correlation coefficient (Pearson’s *r*) was used to investigate the relationship between height and graft dimensions.

A post-hoc power analysis was conducted to confirm the adequacy of the sample size. The mean peripheral diameter of 8.5 ± 0.4 mm was used for this purpose. The anticipated mean peripheral diameter was 8.0 mm, based on a study by Park et al. [16], which demonstrated better outcomes after ACL reconstruction in patients with a graft diameter of 8.0 mm or greater. With an alpha level of 0.05, a minimum sample size of 5 per group was sufficient to achieve a power of 80%.

## Results

### Specimens

Sixteen knees from eight Thiel-embalmed cadavers (4 males and 4 females) were included in this study. The average age at the time of death of the donors was 64.7 ± 9.9 years (ranging from 52 to 81 years). The mean height of the specimens was 159.2 ± 7.9 cm (Table 1).

**Table 1:** Demographic data.

Variable	Mean ± SD
Age, years	64.7 ± 9.9
Gender, male (%)	4 (50)
Height, cm	159.2 ± 7.9

### The graft’s length before folding, mid-diameter after triple folding, peripheral diameter after triple folding, and length after triple folding

The mean graft length before folding was 289.0 ± 10.3 mm (ranging from 240.2 to 277.0 mm). The mean mid-diameter after triple folding was 9.7 ± 0.7 mm (ranging from 8.5 to 10.6 mm). The mean peripheral diameter after triple folding was 8.5 ± 0.4 mm (ranging from 8.0 to 9.5 mm). The mean length after triple folding was 93.1 ± 4.7 mm (ranging from 88.0 to 101.7 mm) (Table 2).

**Table 2:** Graft length before folding, mid-diameter after triple folding, peripheral diameter after triple folding, and length after triple folding. SD, standard deviation.

Variable	Mean ± SD	Range
Graft’s length before folding, mm	289.0 ± 10.3	240.2–277.0
Mid-diameter after triple folding, mm	9.7 ± 0.7	8.5–10.6
Peripheral diameter after triple folding, mm	8.5 ± 0.4	8.0–9.5
Length after triple folding, mm	93.1 ± 4.7	88.0–101.7

### Graft dimensions by gender

The analysis of graft dimensions by gender revealed no statistically significant differences. The graft length before folding was 290.4 ± 10.6 mm in males and 287.5 ± 10.6 mm in females (*p* = 0.589). The mid-diameter after triple folding was 9.7 ± 0.5 mm in males and 9.7 ± 10.1 mm in females (*p* = 0.975). The peripheral diameter after triple folding measured 8.7 ± 0.5 mm in males and 8.4 ± 0.2 mm in females (*p* = 0.107). The length after triple folding was 94.5 ± 5.7 mm in males and 91.7 ± 3.1 mm in females (*p* = 0.235) (Table 3).

**Table 3:** Graft length before folding, mid-diameter after triple folding, peripheral diameter after triple folding, and length after triple folding by gender. SD, standard deviation.

Variable	Male (Mean ± SD)	Female (Mean ± SD)	*P*-value
Graft length before folding, mm	290.4 ± 10.6	287.5 ± 10.6	0.589
Mid-diameter after triple folding, mm	9.7 ± 0.5	9.7 ± 10.1	0.975
Peripheral diameter after triple folding, mm	8.7 ± 0.5	8.4 ± 0.2	0.107
Length after triple folding, mm	94.5 ± 5.7	91.7 ± 3.1	0.235

### Correlation between graft dimension and height

The analysis of the correlation between graft dimensions and height showed weak to moderate correlations for all measured variables; however, none of these correlations were statistically significant. The graft length before folding had a Pearson’s *r* of 0.145 with a *p*-value of 0.591. Similarly, the mid-diameter after triple folding had a Pearson’s *r* of 0.114 with a *p*-value of 0.674. Although the peripheral diameter after triple folding showed a Pearson’s *r* of 0.377, the *p*-value was 0.150. Finally, the length after triple folding exhibited a Pearson’s *r* of 0.399, but the *p*-value was 0.126. Therefore, due to the lack of statistical significance, there was no meaningful linear relationship between height and any of the graft dimensions (Table 4).

**Table 4:** Correlation between graft dimension and height. Pearson correlation coefficient, Pearson’s *r*.

Variable	Correlation with height
Graft length before folding	Pearson’s *r* = 0.145
	*P*-value = 0.591
Mid-diameter after triple folding	Pearson’s *r* = 0.114
	*P*-value = 0.674
Peripheral diameter after triple folding	Pearson’s *r* = 0.377
	*P*-value = 0.150
Length after triple folding	Pearson’s *r* = 0.399
	*P*-value = 0.126

## Discussion

The major findings of this study indicate that the minimally invasive harvesting of the superficial band of the quadriceps tendon resulted in adequate graft dimensions. Gender-based comparisons revealed no statistically significant differences in these dimensions between males and females. Additionally, correlation analysis showed weak to moderate correlations between graft dimensions and height, none of which were statistically significant, indicating no meaningful relationship between height and graft dimensions.

The study by Park et al. showed that the diameter of the hamstring tendon autograft was influenced by several patient-specific factors. While larger graft diameters (≥8.0 mm) correlated with better outcomes, the study did not find significant differences in failure rates based on other variables like BMI, gender, or athletic status. This research suggests the importance of considering graft diameter in ACL reconstructions to potentially reduce failure rates [16]. In this study, the author introduced a minimally invasive technique for harvesting quadriceps tendon autografts, which involved detaching only the superficial layer of the tendon [17]. This method was proposed to have the advantage of preserving quadriceps function postoperatively. However, concerns existed regarding whether this technique would yield an adequate graft diameter for ACL reconstruction. To address these concerns, the author conducted a cadaveric study to evaluate the effectiveness of this technique in producing a sufficient graft diameter. The findings revealed that the mean mid-diameter after triple folding was 9.7 ± 0.7 mm, with graft diameters ranging from 8.5 mm to 10.6 mm. Additionally, the mean peripheral diameter after triple folding was 8.5 ± 0.4 mm, with graft diameters ranging from 8.0 mm to 9.5 mm. These results suggested that the minimally invasive harvesting technique could produce an adequate graft diameter for ACL reconstruction.

Regarding the length of the triple-folded graft for conventional or transtibial ACL reconstruction, the required graft length was approximately 80 mm, with 20 mm on the femoral side, 30 mm on the tibial side, and 27–42 mm within the joint space [18, 19]. To address concerns about the sufficiency of graft length, this study measured the length of the graft after triple folding. The results demonstrated that the mean graft length after triple folding was 93.1 ± 4.7 mm, with lengths ranging from 88.0 mm to 101.7 mm. These findings suggested that the minimally invasive harvesting technique could produce not only an adequate graft diameter but also a sufficient graft length for ACL reconstruction. However, the authors did not investigate the graft diameter for the all-inside technique, as the conventional method already provides an adequate diameter for ACL reconstruction.

The minimally invasive technique for harvesting the superficial layer of the quadriceps tendon offered several advantages [12]. It featured low donor site morbidity due to a small incision, resulting in less postoperative discomfort and a faster recovery [17]. By only sacrificing the superficial layer, the method preserved deeper structures, minimizing overall tissue disruption. Additionally, it did not require special or specific instruments, simplifying the procedure. The graft size and length were predictable, and the foldable nature of the graft allowed for a wider range of fixation techniques. Importantly, this approach eliminated the risk of patellar-associated complications and saphenous nerve injuries commonly associated with bone-patellar tendon-bone and hamstring autografts [6, 20, 21]. Despite its advantages, the harvest of a superficial quadriceps tendon autograft is not without potential drawbacks. Reported complications include anterior knee pain, donor-site morbidity, temporary quadriceps weakness, and hematoma [22]. Careful dissection and proper technique are essential to minimize these risks.

Several autograft options were available for ACL reconstruction, including hamstring, bone-patellar tendon-bone, and quadriceps tendon autografts [23]. Biomechanical studies indicated that hamstring autografts were associated with increased laxity and enlargement of the femoral tunnel aperture over time [24]. Additionally, these grafts were linked to postoperative complications such as hamstring weakness and saphenous nerve injury during harvest [6, 25]. Bone-patellar tendon-bone autografts offered the advantage of robust bone-to-bone healing, yet they also carried risks, including anterior knee pain, patellar tendinitis, patellar fractures, and difficulties with kneeling [26, 27]. Soft tissue quadriceps tendon autografts gained popularity as an alternative, primarily due to their higher collagen content compared to hamstring tendons, which were thought to enhance graft healing [28]. However, the standard quadriceps autograft typically consisted of a single tendon band, limiting the available fixation techniques [12]. This study demonstrated that a minimally invasive technique for harvesting the superficial layer of the quadriceps tendon provided an autograft that, when folded into three layers, achieved sufficient length and diameter for ACL reconstruction. Although a biomechanical study has reported increased laxity and femoral tunnel widening with hamstring autografts [23], these findings are not universally supported. Recent systematic reviews and meta-analyses comparing graft types have shown no consistent superiority in terms of residual laxity or failure rates [29].

However, no clinical outcomes of ACL reconstruction using the superficial band quadriceps tendon autograft have been reported. Therefore, future research should focus on long-term clinical outcomes and compare this technique with other graft types. Additionally, studies involving large sample sizes and diverse patient populations, including variations in age, race, and gender, would help validate the generalizability of this technique.

This study had several limitations. First, being conducted on cadaveric specimens, the findings may not accurately reflect the conditions of living tissue, which could impact their clinical relevance. Second, the mean age of the specimens was 64.7 years, which may not be representative of the broader ACL reconstruction population, particularly as muscle and tendon mass tend to decrease with age. Third, the study was limited to a single institution and exclusively involved Asian samples, which may restrict the generalizability of the findings, as variations in quadriceps muscle and tendon mass across different racial groups could influence the outcomes.

## Conclusion

Minimally invasive harvesting of the superficial band of the quadriceps tendon was found to be feasible for ACL reconstruction, providing adequate graft dimensions. Further clinical studies were recommended to confirm these findings.

## Data Availability

The data that supports the findings of this study are available from the corresponding author upon reasonable request.
